# The impact of COVID-19 on the future of orthopaedic training in the UK

**DOI:** 10.1080/17453674.2020.1795790

**Published:** 2020-07-31

**Authors:** Rupen Dattani, Catrin Morgan, Lily Li, Katharine Bennett-Brown, Rupert M H Wharton

**Affiliations:** Department of Trauma and Orthopaedics, Chelsea and Westminster NHS Foundation Trust, London, UK

## Abstract

The COVID-19 pandemic has had a major impact on global healthcare systems, has drastically affected patient care, and has had widespread effects upon medical education. As plans are being devised to reinstate elective surgical services, it is important to consider the impact that the pandemic has had and will continue to have on surgical training. We describe the effect COVID-19 has had at all levels of training in the UK within trauma and orthopaedics and evaluate how training might change in the future. We found that the COVID-19 pandemic has significantly impacted trainees within trauma and orthopaedics at all levels of training. It had led to reduced operative exposure, cancellations of examinations and courses, and modifications to speciality recruitment and annual appraisals. This cohort of trainees is witnessing novel methods of delivering orthopaedic services, which will continue to develop and become part of routine practice even once the pandemic has resolved. It will be important to observe the extent to which the rapid changes currently being introduced will impact the personal health, safety, and career progression of current trainees.

The COVID-19 pandemic has had a major impact on global healthcare systems, has drastically affected patient care, and has had widespread effects upon medical education. On the 23rd of March 2020, the UK government imposed a lockdown and introduced stringent social distancing measures in response to the rising number of COVID-19 infections. In the field of trauma and orthopaedics (T&O), COVID-19 led to an immediate restructuring of services, redeployment of doctors, and cancellation of elective operating.

As plans are being devised to reinstate elective surgical services in the UK, it is important to consider the impact that the pandemic will have upon surgical training. This article describes the effect COVID-19 has had at all levels of training in the UK within T&O and evaluates how training might change in the future.

## The UK training pathway

Medical students spend between 4 and 6 years of study at medical school before entering a foundation training scheme. This is a 2-year, work-based training programme after which doctors can apply to enter a specialty or general practice training programme. Orthopaedic specialty training is divided into 2 stages, core and higher specialty programmes (Figure). Core surgical training is usually 2 years and its purpose is to allow the trainee to develop basic and fundamental surgical skills common to all surgical specialities, together with some speciality-specific surgical skills. On completion of core training, trainees enter a 6-year specialty training scheme, which is akin to the residency programme in North America.

**Figure:**
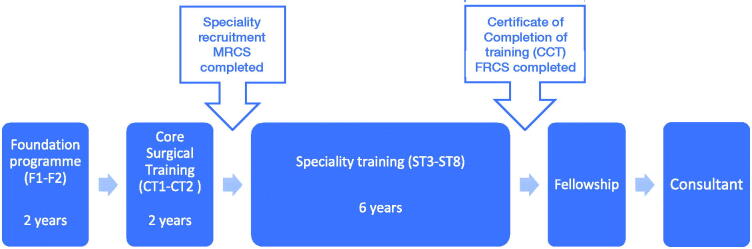
Training pathway for trauma and orthopaedic surgery in the UK.

## Foundation year (FY) doctor experience

There are estimated to be just under 14,000 FY doctors in the UK (The UK Foundation Programme, 2019). There are 6 rotations during foundation training and only 1 of these is in a surgical specialty. For many foundation doctors (FY), this may be the only exposure to surgery prior to choosing a specialty training programme. Those with a rotation in trauma and orthopaedics often already have an interest in the specialty. The rotation gives an invaluable insight into the field of T&O and provides the trainees an opportunity to build their portfolio for the application to core surgical training.

During the early stages of the pandemic, FY doctors were redeployed away from surgical specialties, including T&O, to medical specialties to support the emergency response. As a result, many FY doctors missed out on the opportunity to work within T&O and this may ultimately influence their future career choices (Peel et al. 2018).

## Core surgical trainee (CST) experience

There are currently 1,284 CSTs in the UK (Health Education England 2019). The initial impact of COVID-19 on CSTs was the immediate suspension of all surgical rotations, which resulted in trainees remaining in their current hospital and surgical speciality. This meant that some CSTs did not rotate into T&O and will have missed out on an important training opportunity. During the height of the pandemic, CSTs who remained within T&O were also redeployed to other specialities. This resulted in a significant reduction in surgical exposure and some CSTs may not meet the current minimum requirements to complete core training. At our institution, over a 3-month period (March 23–June 23, 2020), CSTs reported a 76% reduction in logbook numbers compared with the same timeframe in 2019.

## Speciality registrars (StR) experience

There are currently 1,158 StRs enrolled in the UK T&O training programme (Joint Committee on Surgical Training 2019). In many institutions, an emergency COVID rota was introduced and StRs became “first on call” for emergency referrals and looking after inpatients. In some regions, StRs were also redeployed to intensive care units.

With the introduction of the national lockdown, there was a dramatic reduction in the number of trauma cases (Hampton et al. 2020). This, coupled with advice encouraging non-surgical management of trauma, has resulted in a significant reduction in trauma operating (British Orthopaedic Association 2020a). Furthermore, where cases have required surgery, there has been an emphasis towards consultant-led operating in order to minimise operative time. All these factors have resulted in a substantial reduction in surgical exposure to orthopaedic trauma. At our institution, over a 3-month period (March 23–June 23, 2020), StRs have reported a 90% reduction in numbers of trauma case operations compared with the same timeframe in 2019. Similar reduction in trainee operating volume has also been reported in other parts of the world during the current pandemic (Jones et al. 2020).

As in most parts of the world, elective orthopaedic surgery in the UK almost completely stopped. Consequently, trainees have not had any elective surgical exposure since the middle of March 2020. A significant component of the orthopaedic curriculum is focused on achieving competencies through the use of the online Intercollegiate Surgical Curriculum Programme (ISCP) and electronic surgical logbook. There are a number of indicative procedures required for the Certificate of Completion of Surgical Training (CCST): a minimum requirement of 1,800 cases with 70% of these as the primary surgeon. As a result of reduced surgical exposure as outlined above, many trainees may fail to achieve the minimum requirements to progress to the next stage of their training.

## Fellowship experience

In an era of increasing subspecialisation, fellowship has become an essential component of orthopaedic education and a prerequisite to application for consultant posts. It is estimated that approximately 90–95% of UK StRs will undertake a specialist fellowship after award of CCST (Ruddell et al. 2018). Many current UK fellows have been redeployed to their home deaneries to support the local COVID-19 response. For those who still remain in their posts, both in the UK and abroad, surgical exposure has reduced dramatically due to suspension of elective services. Consequently, many fellows may feel they have not gained enough experience to transition to consultant grade and may decide to do an additional fellowship to gain more exposure and further develop their subspecialty skills.

Many trainees who were due to commence fellowships have had to delay the start of their training until elective orthopaedic services resume. For others, even though units were keen to receive them, trainees were apprehensive about starting in hospitals given the constant fear of sickness or redeployment whilst living far from home. For those hoping to start fellowships abroad, the closure of international borders has created additional uncertainty.

Finally, COVID has struck at the time of year when most fellows are either finalising their future employment or have already committed to consultant jobs. The uncertainty surrounding future services as a result of the pandemic has placed additional anxiety and apprehension on this cohort of trainees.

## The future of orthopaedic training post-COVID

The impact of COVID-19 on orthopaedic training in the UK will be felt for months and possibly years to come. This will necessitate adaptability and restructuring of training programmes to allow trainees access to the learning opportunities that they require to progress through their surgical career. Some of these changes may need to be temporary to ensure current trainees progress through their training schemes, whilst other changes will need to be long term and in preparedness for a possible second wave of COVID-19 infections. From recruitment to completion of training and beyond, educational modalities are likely to change with positive and negative effects associated with all aspects.

### Training structure

At the time of writing (July 7, 2020), the first peak of virus infections was diminishing and it remains to be seen whether further peaks will necessitate alternative plans. Currently, the British Orthopaedic Association (BOA), following guidance from NHS England, is devising plans to allow elective services to resume (British Orthopaedic Association. 2020b). In order to ensure patient and staff safety, it will be essential to develop COVID-free “green” pathways to deliver elective orthopaedic services (British Orthopaedic Association 2020b). This may lead to the separation of trauma training from elective orthopaedic training to minimise cross-contamination. It is difficult to predict whether this change is short term, requiring readjustment of the normal process, or is long term, necessitating the need for restructuring of the training programme. This will have implications for the management of the on-call system as those involved in planned care will not be able to be part of the on-call rota. Trainees will have to ensure that their trauma skills do not decline during elective periods and further reassessment prior to CCST may be required to ensure day one consultants remain able to deliver a safe orthopaedic trauma service.

### Speciality recruitment and Annual Review of Competency Progression

In the UK, the traditional national recruitment process for orthopaedic surgeons takes place every April; this year it was cancelled due to the COVID-19 pandemic. A contingency plan was put in place that did not include a formal interview process. Instead, national training numbers were allocated using a unvalidated self-assessment score, which ranked the trainees. This process was poorly received by applicants.

With social distancing measures likely to stay in place for the foreseeable future, it is possible that virtual interviews may become the norm. A streamlined virtual interview process using a centralized virtual interview platform or the use of a virtual interview as a screening tool for face-to-face interviews would be possible methods by which recruitment could proceed in the future (Jones and Abdelfattah 2020). Furthermore, an assessment of how implementation of videoconferencing technology in the orthopaedic matching scheme may affect the selection process will need to be undertaken from both a programmatic and candidate standpoint (Jones and Abdelfattah 2020).

It may still be possible to keep the current format of the recruitment process and assess prospective candidates via a virtual interview process using online methods, using this as an opportunity to review and improve the interview process for future trainees. Such platforms have been shown to be successful for surgical training programmes in the USA during the current pandemic (Day et al. 2020). CSTs may lack experience and skill with virtual interviews given that this is not a widely used method of assessing prospective trainees; teaching of such skills may therefore need to be incorporated into future training programmes. Some institutions have offered online interviews for recruitment into other specialities and have shared their experience on how trainees can best show their interpersonal skills during this process (Jones and Abdelfattah 2020).

The Annual Review of Competency Progression (ARCP) process is the means by which training CSTs and StRs are reviewed each year to ensure that they are offering safe, quality patient care, and to assess their progression against standards set down in the curriculum for their training programme. The ARCP process is conducted through face-to-face meetings with a panel consisting of the training programme director and at least 2 other surgeons. Although these could recommence with appropriate social distancing measures in place, it is likely that these will become virtual assessments in the future. The quality of the ARCP process, whether performed as a face-to-face meeting or virtual assessment, is unlikely to be affected as trainees will still have an opportunity to raise any issues and receive feedback from the panel.

### Clinics

With the introduction of social distancing measures there has been a massive expansion in the use of telemedicine and video-assisted consultations during the COVID-19 crisis. Numerous medical schools in the USA have incorporated telemedicine training into the preclinical undergraduate curriculum (Waseh and Dicker 2019). The continued use of telemedicine in the UK is likely to become part of normal clinical practice in the future, especially for patients who cannot easily attend face-to-face consultations or patients deemed to be at high risk from COVID-19. Orthopaedic trainees will need to receive formal training to use telemedicine in a professional, safe, and evidence-based manner. Telemedicine competencies may need to be built into existing components of the orthopaedic curriculum.

Physical examination of patients in clinics forms a crucial part of training. With the increased use of telemedicine, modified interactive orthopaedic examination techniques have been developed and trainees will need to become familiar with such virtual examinations (Tanaka et al. 2020). Despite this a thorough clinical examination will not be possible. Face-to-face consultation is fundamental in every doctor–patient relationship and it remains to be seen whether telemedicine will equally allow such trusting relationships to be built. In the future, virtual clinics may be useful as an initial screening consultation during which a pertinent clinical history can be taken, thus minimising the length of face-to-face consultations. In addition, remote clinics may also have a place in the assessment of postoperative patients where they have been shown to be a safe and a cost-effective means of delivering patient care (Parisien et al. 2020, Buvik et al. 2019, Goldstein et al. 2019, Tanaka et al. 2020).

## Surgical skills

In the short term, it is likely that there will be fewer cases being done on each operating list and hence there will be a reduction in operative training opportunities. In the longer term, with an increased backlog of cases, it is plausible there may be more operating lists and therefore training opportunities could improve. In order to optimise surgical skills, simulation may play an important role in preparing a trainee for performing an operation for the first time, as well as being deployed alongside operating to practise newly acquired skills. The use of simulation training has been shown to reduce intraoperative complications in other surgical specialities (Staropoli et al. 2018). In T&O, it has been demonstrated to be effective in skill acquisition for shoulder and knee arthroscopy (Aim et al. 2016, Bartlett et al. 2018) as well as wrist fracture reduction (Jackson et al. 2020).

Training on a virtual simulator can be realistic and the ability to perform surgical steps countless times without additional cost per attempt makes this an attractive training tool. Another advantage of simulation training is that skills can be measured and evaluated in a standardized manner. Virtual reality training has been shown to improve technical skills in orthopaedic surgery and may need to be integrated into the orthopaedic curriculum in the UK, following in the steps of other surgical specialities (Aim et al. 2016, Staropoli et al. 2018).

The COVID-19 pandemic may act as a catalyst for the use of augmented reality (AR) and immersive simulation within orthopaedic surgery. Although AR is a field in its infancy, it has been shown to have a wide variety of applications in both elective and trauma surgery. AR enables hands-free real-time access to operating room resources, helping promote telemedicine and education (Laverdiere et al. 2019). It has been used as a platform that allows a surgeon to deliver virtual assistance remotely to another surgeon by layering a live video of their hands reaching into the local surgeon’s operative field in real time, to provide complex visual instructions within shoulder arthroscopy (Ponce et al. 2014). Its use could be particularly valuable for surgical education during times of limited operating combined with the benefit of reducing the number of surgeons in the operating theatre and hence reducing virus transmission risk. Immersive simulation has also been shown to improve translational technical and non-technical skill acquisition over traditional learning in orthopaedic residents in cadaveric shoulder surgery (Lohre et al. 2020).

### *Examination*s

The Fellowship of the Royal College of Surgeons (FRCS) examination undertaken by trainees in the UK and Ireland has served as a benchmark for the standard required of a Day 1 Consultant Orthopaedic Surgeon in a District General Hospital. Currently, all examinations have been suspended pending a review of capacity for delivery of these examinations effectively and safely during the pandemic.

The FRCS (Tr &Orth) examination encompasses 2 sections: section 1 is the written exam and section 2 the clinical exam. Section 1 of the examination is already conducted remotely using computer-based tests held at local driving test centres throughout the UK and Ireland. This part of the exam could easily be reintroduced as long as appropriate social distancing measures are implemented at testing centres. Other Royal Colleges are moving towards a new method of online proctored exam delivery where the written component of the examination can take place in the candidate’s own home or workplace (Royal College of Opthalmologists 2020).

Section 2 of the examination consists of the clinical short and long cases and structured oral interviews. Reintroduction of the clinical section will prove difficult to deliver as candidates, examiners, and patients will need to wear personal protective equipment (PPE) during the process. Clinical examinations are usually held in hospitals and it would be unethical to ask patients to attend institutions where they may be at a higher risk of exposure to COVID-19. In the short term, with appropriate planning, the venue could be changed to a hotel or conference centre with a smaller number of candidates sitting the examination to minimise risk to the patient. The use of virtual patients has previously been explored as a method for creating high-fidelity simulated patient interactions that can overcome many of the challenges associated with using live standardised patients (Triola et al. 2006). The virtual patient has the advantage of being easily modified to demonstrate a variety of clinical scenarios and has successfully been used to assess clinical competencies (Botezatu et al. 2010).

The oral component of the examination consists of four 30-minute patient-based scenarios. With appropriate planning and validation, it may be possible to conduct this part of the assessment remotely. Similar types of oral examinations have previously been assessed using telemedicine technology while giving formative feedback in a way that is financially feasible for the organisers and well received by candidates (Hannon et al. 2020).

### Trauma meetings

Most orthopaedic departments across the UK hold a morning trauma meeting, reviewing acute trauma admissions to discuss their management. With the introduction of social distancing measures, many orthopaedic departments have implemented a remote “tele-conference” system with a screenshot broadcast of presented radiographs (Pearce et al. 2020). This avoids the potential risk of transmission within the orthopaedic workforce as well as facilitating access to the trauma meeting for those in self-isolation or those who have been advised to shield. With social distancing measures likely to stay in place for the foreseeable future, the use of virtual trauma meetings is likely to continue and may even become part of normal orthopaedic practice in the post-COVID era.

### Teaching, courses, and conferences

Most orthopaedic rotations hold weekly regional teaching designed to cover the syllabus for all trauma and orthopaedic topics to FRCS level. Currently, teaching in most rotations has stopped and will most likely resume in the form of virtual teaching with the use of tele-conferencing. The use of online platforms to deliver speciality teaching has been shown to be well received by trainees, and can result in high attendance rates and improved success in postgraduate examinations (MacDonald et al. 2013, Smith et al. 2013).

Orthopaedic journal clubs form a crucial part of orthopaedic training and are important in educating trainees in the skills of critically appraising scientific papers. As with regional teaching, journal clubs are likely to move to an online platform in the future. Virtual journal clubs can have many advantages including improved trainee participation as well as maintenance of an electronic record of the discussion threads, the cases discussed, and the papers flagged for future review, which can easily be accessed by all trainees at a later date (Palan et al. 2012). With trainees being able to attend virtually, this means less time away from family and a step in the right direction in improving a surgeon’s work/life balance with no detrimental effects to educational benefits.

Attendance at courses is an important component of orthopaedic training and is one of the mandatory elements assessed at the final ARCP (Joint Committee on Surgical Training. 2017). At present, all courses have been cancelled and there has been a rapid expansion of web-based learning resources with increased availability of online lectures and webinars (Plancher et al. 2020). Even before the COVID crisis, web-based teaching and video-based surgical learning had gained popularity among trainees and had been shown to be an effective education tool (Rogers et al. 2019). Such means of learning are likely to become an essential method of delivering orthopaedic education even after the current pandemic has been resolved.

Attending regional and national orthopaedic conferences also forms an important part of orthopaedic training. Even after the post-COVID era, organisers of these meetings may feel pressure to change to virtual platforms. Web-based meetings are becoming increasingly popular as in-person meetings can be costly, time-intensive, and involve time away from home (Moran et al. 2018). Some emerging platforms have interactive components such as chat and messaging whereby attendants can interact actively with presenters in a similar manner to the traditional question-and-answer periods. Until we find better ways of networking virtually, trainees will not have the benefit of this type of mentorship without in-person meetings.

## Conclusion

The COVD-19 pandemic is an evolving crisis that has had a profound impact on global healthcare systems. Along with many in society, the impact on orthopaedic training will have far-reaching implications, hence the term the “COVID Generation”. As an immediate response to the pandemic, this cohort of trainees have had to adapt quickly to using novel methods of delivering orthopaedic services. It will be important to observe the extent to which the rapid changes will impact the personal health, safety, and career progression of current trainees. Looking to the future, this pandemic has provided a unique opportunity for educators to change the way surgical training has traditionally been delivered and to help shape the future of medical education.

There are numerous examples (e.g. HIV, wars, terrorist attacks) whereby learning from challenging situations has helped transform science and healthcare. Isaac Newton revolutionised the scientific world while in isolation during the plague. Benjamin Franklin once said, “Out of adversity comes opportunity”. All medical trainees now have the opportunity to help make seminal changes in healthcare, and for orthopaedic trainees this is their time to contribute to the advancement of their specialty.

## Funding and conflicts of interest

No funding was received in connection with this article. The authors have no conflicts of interest to declare.
